# How Imported Pathology Becomes Overlooked as Resources Increase: An Example of a Neglected Tropical Disease (NTD)

**DOI:** 10.3390/tropicalmed11080234

**Published:** 2026-08-20

**Authors:** Fernando de la Calle-Prieto, Lucía Platero, Jorge Ligero-López, Marta Díaz-Menéndez, Guillermo Ruiz-Carrascoso

**Affiliations:** 1 National Referral Unit for Imported Diseases and International Health, High Level Isolation Unit, La Paz-Carlos III University Hospital, 28046 Madrid, Spain; 2 CIBERINFEC (Center for Biomedical Research in Infectious Diseases Network), 28029 Madrid, Spain; 3 La Paz University Hospital Research Institute (IdiPAZ), 28046 Madrid, Spain; 4 Department of Internal Medicine, La Paz-Carlos III University Hospital, 28046 Madrid, Spain; 5 Department of Microbiology, San Jorge University General Hospital of Huesca, 22004 Huesca, Spain; 6 Clinical Microbiology Department, La Paz-Carlos III University Hospital, 28046 Madrid, Spain

**Keywords:** loiasis, neglected tropical diseases, imported infections, filariasis, migrant health, diagnostic delay

## Abstract

This case report highlights how neglected tropical diseases (NTDs) may remain underdiagnosed in high-resource settings despite advanced healthcare systems. We describe an 18-year-old woman from Equatorial Guinea living in Spain who presented with chronic pruritus and episodic ocular symptoms and was ultimately diagnosed with ocular loiasis after the incidental extraction of a conjunctival filaria. Diagnostic evaluation included microscopy, serology, and molecular techniques, confirming *Loa loa* and *Mansonella perstans* co-infection alongside evidence of exposure to other parasitic infections. Management involved staged antiparasitic therapy with albendazole, ivermectin, praziquantel, and diethylcarbamazine, combined with corticosteroids to mitigate treatment-related reactions. The patient showed clinical improvement, although follow-up was irregular and microfilaremia persisted. This case illustrates key challenges in non-endemic settings, including low clinical suspicion, fragmented care pathways, and barriers to healthcare access among migrant populations. Despite the availability of sensitive diagnostic tools, delayed recognition remains common when tropical diseases are not considered in the differential diagnosis. Comprehensive, multidisciplinary approaches are essential, particularly in patients from endemic regions who may harbor multiple co-infections. Strengthening clinical awareness, referral pathways, and appropriate screening for NTDs may help reduce diagnostic delays and improve patient outcomes in increasingly globalized healthcare settings.

## 1. Introduction

Filarial infections represent a significant health concern in endemic regions of Central and West Africa [1,2,3]. These parasitic diseases are caused by nematodes of the superfamily *Filarioidea*, among which *Loa loa* and *Mansonella perstans* are particularly relevant because of their co-endemicity with other filarial infections, such as onchocerciasis and lymphatic filariasis [4,5]. These infections can lead to severe adverse reactions when antiparasitic treatment is administered without an appropriate diagnosis. Diagnostic methods have evolved from traditional microscopy to molecular techniques and biomarkers [6,7]. However, these diseases may paradoxically remain underdiagnosed in healthcare systems with greater economic and technological resources because of low clinical suspicion. Although microscopy remains a fundamental diagnostic tool, molecular and serological techniques can provide greater sensitivity, despite limitations in specificity. Combining these methods may improve diagnostic accuracy in both endemic and non-endemic settings and facilitate appropriate disease management [7,8,9].

The identification of microfilariae in stained blood smears (Giemsa or hematoxylin-eosin) remains widely used. However, sensitivity is limited in patients with low parasite burdens or amicrofilaremia. Techniques such as Millipore membrane filtration or sedimentation can increase microscopic sensitivity, although they still depend on the presence of microfilariae in the sample [2,6].

Polymerase chain reaction (PCR) is a highly sensitive and specific technique for detecting filarial DNA in blood and can identify infections in amicrofilaremic individuals. Among molecular methods, real-time PCR enables quantification of *Loa loa* and *Mansonella perstans* DNA, facilitating infection monitoring. Additionally, cytochrome oxidase subunit I PCR (COI-PCR), targeting mitochondrial regions, provides a reliable method for species identification [1,4,6].

Antibody detection using ELISA and immunoprecipitation techniques has identified immunoglobulin G4 (IgG4) as a marker of active *Loa loa* infection. Likewise, PEG-ELISA has shown diagnostic potential, although its specificity may be limited by cross-reactivity with *Mansonella perstans* [2,3,10].

## 2. Case Presentation

We report the case of an 18-year-old woman from Equatorial Guinea who has lived in Spain since May 2022. Her medical and family history was unremarkable. She reported no known allergies, regular medication use, substance use, high-risk sexual behavior, or animal contact. She lived in a landscaped residential area on the outskirts of Madrid.

Since the age of 12, the patient had experienced intermittent pruritus and transient inflammatory and burning skin lesions that resolved spontaneously within hours. She reported no fever, arthralgia, chest pain, respiratory symptoms, abdominal pain, changes in bowel habits, dysuria, hematuria, or other systemic manifestations. She had also experienced episodes of painless red eye associated with a foreign body sensation, which she described as a “moving thread,” without visual disturbances.

**Figure 1:**
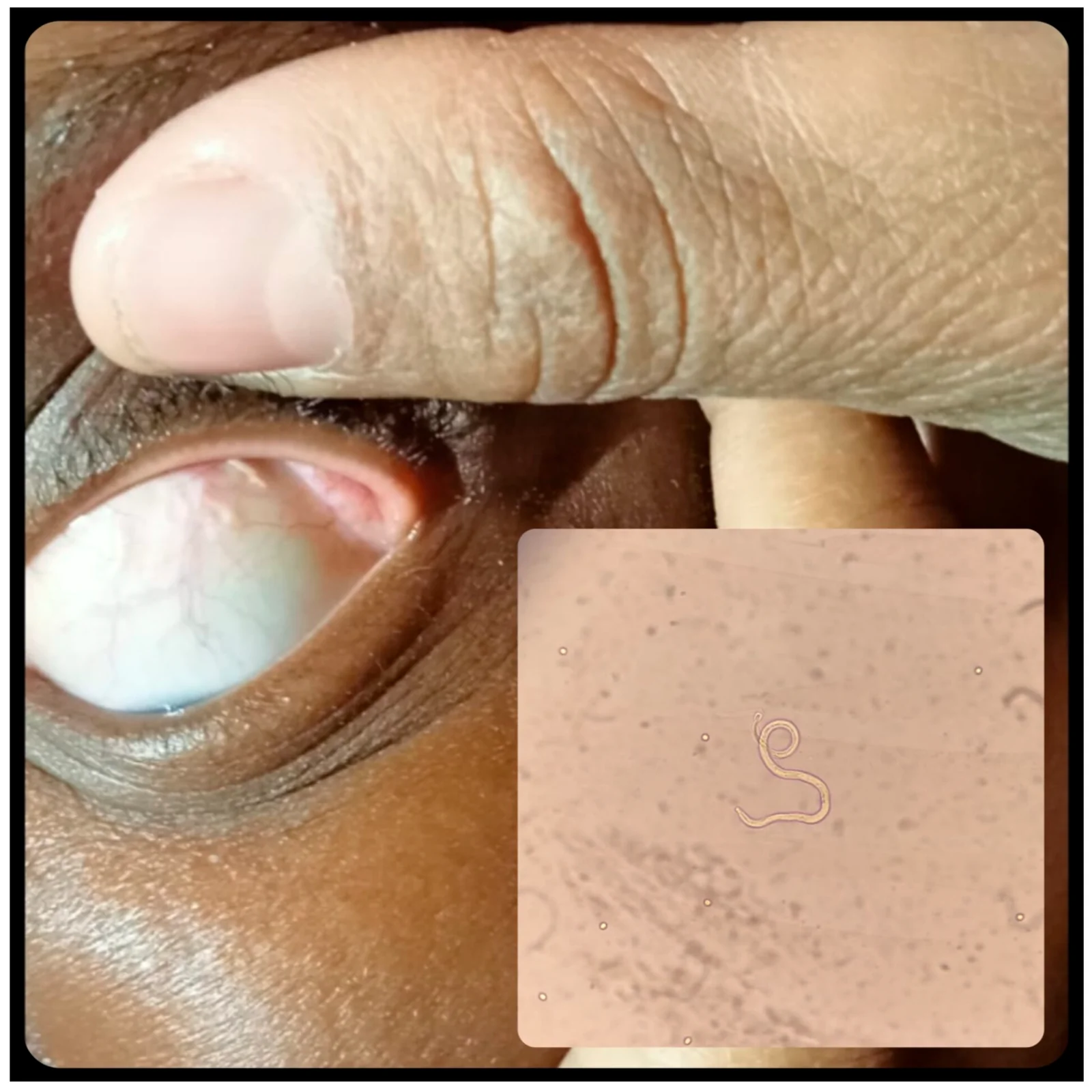
Observation of ocular migration of *Loa loa* on the day of presentation to the Emergency Department, documented through ophthalmologic photography. In the inset, the specimen extracted at that moment and sent to the Microbiology Laboratory, where the extracted filaria was observed under microscopy.

After attending several healthcare facilities over an eight-month period without a definitive diagnosis or specific diagnostic testing, she presented to the Emergency Department. Ophthalmological evaluation identified a thread-like foreign body in the eye, which was subsequently extracted (Figure 1). Fundoscopy findings were normal. The patient was informed that no further intervention or follow-up was required after removal of the foreign body.

The extracted material was sent to the Microbiology Department of Hospital Universitario La Paz-Carlos III in Madrid, the National Reference Center for Imported Pathology, which provides 24/7 microbiology services. Identification of the ocular filaria prompted referral to our Imported Pathology and International Health Unit.

Our initial evaluation considered *Loa loa* filariasis, with *Onchocerca volvulus* and *Mansonella streptocerca* included in the differential diagnosis [1,2]. Other parasitic infections, such as cysticercosis, which involves the eye in approximately 20% of cases, were also considered. Alternative causes of painless red eye, including foreign bodies, bacterial or viral conjunctivitis, and chemical, physical, or allergic keratitis, were considered less likely given the bilateral and chronic nature of the symptoms.

Physical examination revealed no Calabar swellings, skin nodules, depigmentation, papules, rash, or other dermatological abnormalities.

Blood tests revealed microcytic hypochromic anemia (hemoglobin, 11.7 g/dL) with iron deficiency and eosinophilia (13.7%; absolute count, 1130). Coagulation parameters, electrolytes, renal and liver function tests, and other laboratory findings were within normal limits, except for a C-reactive protein (CRP) level of 0.8 mg/dL. Comprehensive microbiological testing based on the patient’s epidemiological background was negative for HIV, syphilis, hepatitis A, B, and C, rickettsiosis, toxocariasis, and *Plasmodium*. However, serological testing was positive for *Strongyloides* and *Schistosoma* IgG, whereas stool and urine parasitological examinations were negative. Assessment of immunity to vaccine-preventable infections showed negative IgG for measles and varicella, positive IgG for hepatitis A, and negative anti-HBs antibodies.

**Figure 2:**
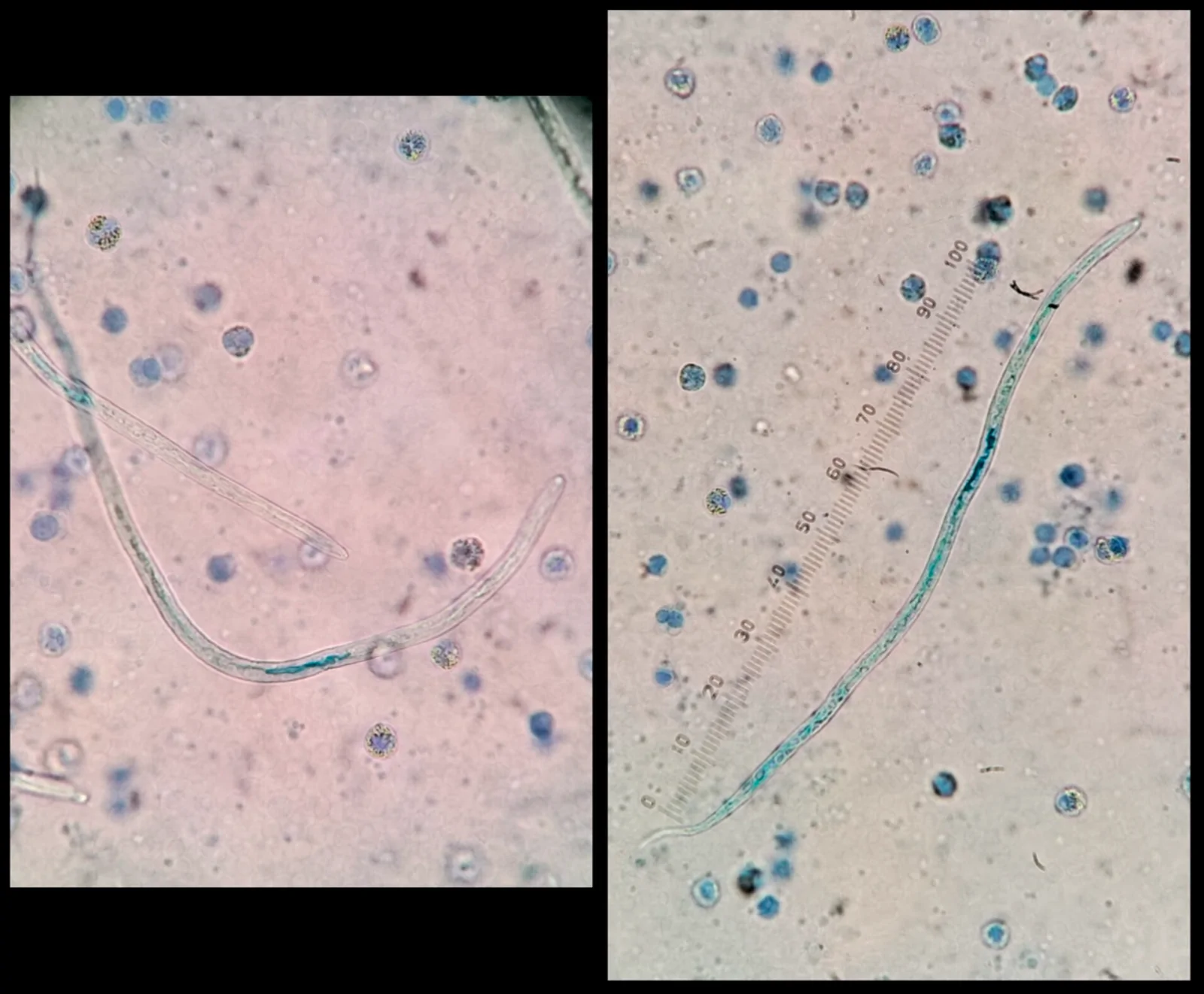
Detailed microscopic examination of the patient’s blood samples, revealing circulating microfilaremia. Original magnification ×400. Scale bar = 250 µm.

Microscopic examination identified the conjunctival foreign body as *Loa loa* (Figure 1). Examination of peripheral blood, collected during the known period of diurnal microfilaremia of *Loa loa*, also revealed microfilariae (Figure 2); however, microfilarial density was not reported by the laboratory at this stage.

**Figure 3:**
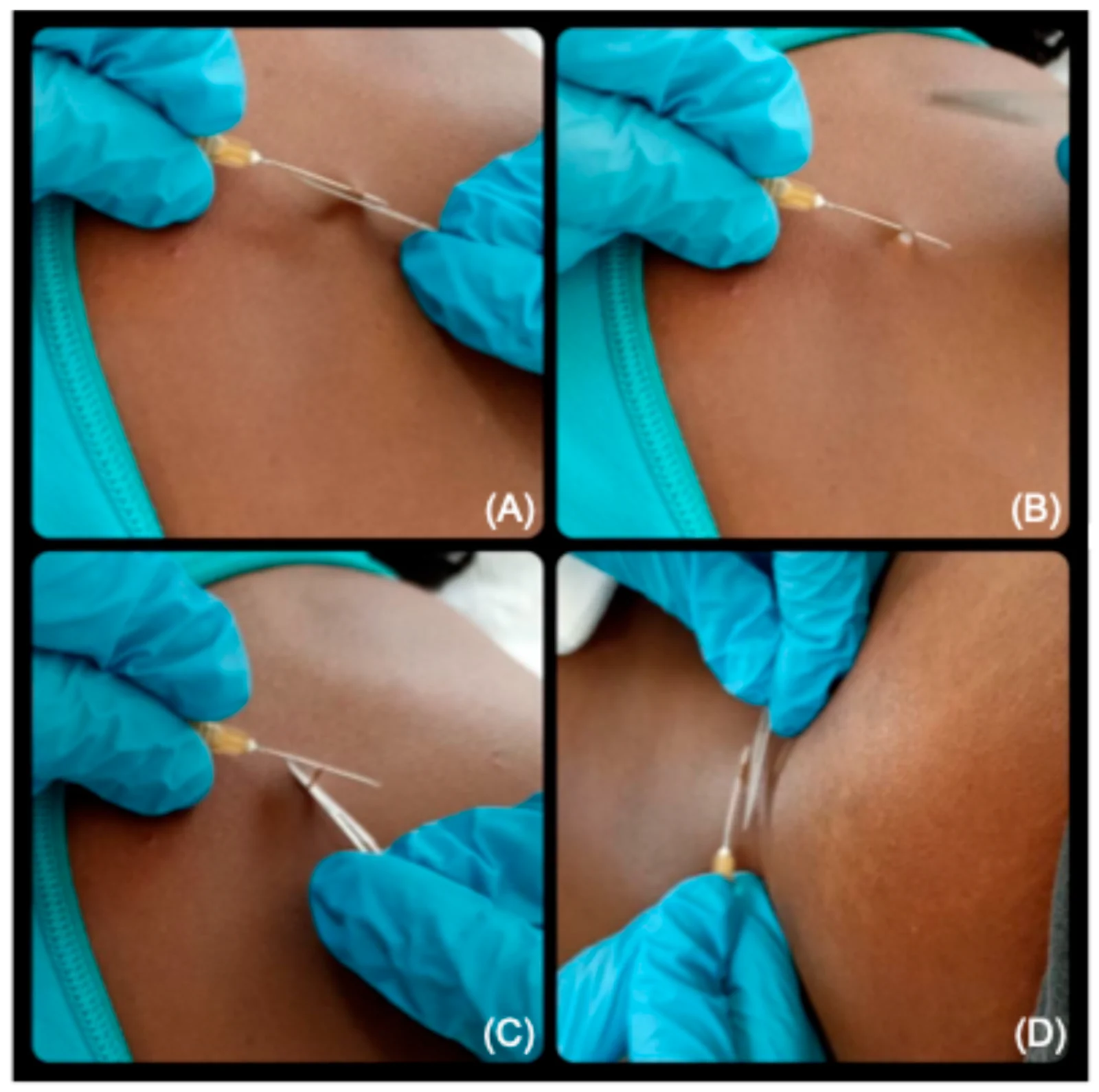
Collection of skin samples for the diagnosis of this patient to rule out other filariases: Panels (**A**–**C**) show the collection of a skin sample from the subscapular area, where superficial skin material should be obtained without blood residues. Panel (**D**) details the sample collection from the patient’s gluteal area.

Further molecular testing of peripheral blood detected *Loa loa* and *Mansonella perstans* DNA, whereas PCR for *Onchocerca volvulus* was negative. Subscapular and gluteal skin biopsies were also performed to screen for other filarial infections, with negative results by both direct examination and PCR (Figure 3).

Following completion of the ophthalmological evaluation and exclusion of *Onchocerca volvulus* infection, treatment for filariasis was initiated. As the exact microfilarial density was not yet available, albendazole was initially administered to reduce the potential microfilarial burden before diethylcarbamazine (DEC), thereby minimizing the risk of severe inflammatory reactions. Although doxycycline targeting *Wolbachia* could have been considered for *Mansonella perstans*, albendazole was preferred because of its additional activity against *Loa loa* microfilaremia.

The patient discontinued follow-up because of concerns that her insurance might require payment for medical care at our center. When she returned several weeks later, she was asymptomatic, with no dermatological manifestations. Follow-up blood tests showed persistent eosinophilia (12%; absolute count, 560), while microbiological analysis confirmed persistent *Loa loa* microfilaremia (4000 microfilariae/mL).

Given the patient’s irregular follow-up, the therapeutic strategy was reassessed. Priority was given to shorter treatment regimens targeting the suspected concomitant helminth infections while further reducing the microfilarial burden before definitive DEC therapy. Ivermectin was selected for its activity against both *Loa loa* and *Strongyloides stercoralis*, followed by weight-adjusted praziquantel for suspected schistosomiasis. After completing these shorter courses without adverse reactions or new symptoms, and once adherence had been established, definitive DEC treatment was initiated with gradual dose escalation to 100 mg every 8 h for 21 days. To reduce inflammatory reactions associated with parasite death, prednisone was administered in a tapering regimen during the first 10 days of DEC treatment.

The patient’s ocular symptoms improved, with resolution of pruritus and foreign body sensation. Following completion of antiparasitic therapy, vaccination was recommended for vaccine-preventable infections for which serological testing was negative.

## 3. Discussion

NTDs remain a major yet under-recognized global health challenge, affecting more than one billion people worldwide. Because of their prolonged latency and persistent parasitism, many NTDs may remain undetected for months or even years after individuals leave endemic regions. With increasing globalization, migration, and international travel, these diseases are increasingly encountered in high-income countries, yet they may be overlooked even where healthcare resources are abundant.

Recognition of NTDs in non-endemic settings is essential, as they may present late or coexist with other parasitic infections, requiring a comprehensive diagnostic and therapeutic approach. In our patient, serology was positive for *Strongyloides stercoralis* and *Schistosoma* spp., whereas stool culture for *Strongyloides* and stool and urine microscopy for *Schistosoma* eggs were negative. Because serology cannot reliably distinguish past from active infection and given the patient’s epidemiological background and absence of previous antiparasitic treatment, these findings were managed as potentially active infections following an individualized risk–benefit assessment. This illustrates a broader challenge in NTDs, where clinical decisions may rely on diagnostic tools with limited ability to distinguish active from previous infection [7,8,9,10].

Evaluation of patients from endemic regions often requires a comprehensive assessment beyond the presenting complaint, including screening for concomitant parasitic infections and vaccine-preventable diseases when appropriate. However, barriers to specialist referral and limited expertise in tropical medicine may delay diagnosis and appropriate management, even in well-resourced healthcare systems [7,8].

The present case exemplifies these challenges. Despite multiple medical consultations over an eight-month period, the diagnosis was established only after ophthalmological evaluation supported by specialized microbiological testing. During this time, the patient remained symptomatic despite the availability of appropriate diagnostic and therapeutic resources.

This delay is not an isolated occurrence. In some settings, limited training in tropical diseases and the absence of clear referral pathways to specialized centers contribute to underdiagnosis. Beyond the impact on individual patients, delayed recognition and treatment of imported infections may also have broader public health implications. Climate change, increasing international mobility, and the expansion of competent arthropod vectors in parts of Europe raise concerns about the potential emergence of local transmission cycles for imported vector-borne diseases. Although sustained local transmission of loiasis has not been documented in Europe, failure to promptly identify imported cases underscores the need for appropriate surveillance, greater clinical awareness, and effective referral pathways to reduce future public health risks [9,10].

A retrospective study from Belgium showed that imported filarial infections, including *Loa loa* and *Mansonella perstans*, were frequently misdiagnosed or diagnosed late, even in referral centers. Similarly, a 25-year review found that many cases of loiasis in non-endemic countries were diagnosed only after complications had developed. These findings highlight persistent challenges in the timely recognition and management of imported tropical diseases in non-endemic settings [1,9].

Imported NTDs disproportionately affect migrant and other vulnerable populations, who may face barriers to timely specialist referral and continuity of care, even within well-resourced healthcare systems. These challenges may affect not only diagnosis but also subsequent therapeutic management. This case illustrates the complexity of managing imported NTDs in clinical practice, where therapeutic decisions must balance parasite burden, concomitant infections, treatment-related risks, and the likelihood of maintaining adherence despite interrupted follow-up. Consequently, management may require individualized strategies rather than rigid protocol-based approaches, underscoring the value of specialized referral units with expertise in tropical medicine and imported infectious diseases [9,10].

Although microscopy remains fundamental for diagnosing filarial infections, its sensitivity may be limited in patients with low parasitemia or amicrofilaremia. Molecular techniques such as PCR and LAMP (Loop-mediated isothermal amplification) can improve diagnostic sensitivity and species identification. However, as this case illustrates, access to advanced diagnostic tools alone is insufficient when clinical suspicion is low or appropriate referral pathways are lacking.

From a public health perspective, delayed recognition of imported NTDs in high-income countries may undermine global elimination efforts. As emphasized by The Lancet Commission on NTDs, sustainable control requires not only interventions in endemic regions but also greater clinical awareness and diagnostic capacity in non-endemic settings [11,12].

This case further highlights the importance of a syndromic approach when evaluating patients from endemic regions. In addition to loiasis, the patient had positive serology for *Schistosoma* spp. and *Strongyloides stercoralis*, illustrating the overlap of helminth infections that may be overlooked when diagnostic strategies focus solely on the presenting complaint. Beyond diagnosis, imported NTDs may require individualized therapeutic planning that accounts for concomitant infections, treatment-related risks, and factors affecting adherence and continuity of care. This complexity underscores the value of multidisciplinary referral units with expertise in tropical medicine, where clinical decision-making can integrate parasitological knowledge, evidence-based management, and patient-specific circumstances.

## 4. Conclusions

This case demonstrates how imported NTDs may remain unrecognized for prolonged periods, even in highly resourced healthcare systems, because of limited clinical awareness and fragmented care pathways. Timely diagnosis, however, is only the first step. Successful management also requires individualized therapeutic strategies that account for parasitological complexity, concomitant infections, treatment-related risks, and factors affecting adherence and continuity of care. In this context, specialized referral units for imported infectious diseases can integrate multidisciplinary expertise, evidence-based management, and patient-centred care, improving clinical outcomes while strengthening preparedness for emerging tropical diseases in non-endemic settings. Ultimately, as global mobility, environmental change, and the expansion of competent vectors reshape the epidemiology of infectious diseases, strengthening the capacity to recognize and manage imported NTDs should be considered both a clinical priority and an important component of global health preparedness.

## Data Availability

The original contributions presented in this study are included in the article. Further inquiries can be directed to the corresponding author.

## Abbreviations

The following abbreviations are used in this manuscript:

NTD	Neglected tropical diseases
PCR	Polymerase Chain Reaction
qPCR	Real-time Polymerase Chain Reaction
COI-PCR	Cytochrome oxidase subunit I Polymerase Chain Reaction
LAMP	Loop-mediated isothermal amplification
DBS	Dried blood spot
IgG4	Immunoglobulin G4
CRP	C-reactive protein
DEC	Diethylcarbamazine
